# Radical Mediated Rapid *In Vitro* Formation of *c*-Type Cytochrome

**DOI:** 10.3390/biom12101329

**Published:** 2022-09-20

**Authors:** Sheikh Muhammad Ibrahim, Sami Ben Aoun, Hiroshi Nakajima, Fethi Kooli, Yoshihito Watanabe

**Affiliations:** 1 Chemistry Department, Faculty of Science, Islamic University of Madinah, Al-Madinah Al-Munawarah 42351, Saudi Arabia; 2 Department of Chemistry, Faculty of Science, Taibah University, Al-Madinah Al-Munawarah P.O. Box 30002, Saudi Arabia; 3 Graduate School of Science, Osaka City University, Osaka 558-8585, Japan; 4 Department of Chemistry, Graduate School of Science, Nagoya University, Nagoya 464-8602, Japan or; 5 Research Center of Materials Science, Nagoya University, Nagoya 464-8602, Japan

**Keywords:** cytochrome *c*_552_, thermally tolerance cytochrome, thiol-ene reaction, Zn-protoporphyrin(IX), sulfuryl radical, Mn-protoporphyrin(IX), *c*-type protein

## Abstract

A cytochrome *c*_552_ mutant from *Thermus thermophilus* HB8 (*rC*_552_ C14A) was reported, where the polypeptide with replaced Cys14 by alanine, overexpressed in the cytosol of *E. coli*. The apo-form of the C14A mutant (apo-C14A) without the original prosthetic group was obtained by simple chemical treatments that retained compact conformation amenable to reconstitution with heme *b* and zinc(II)-protoporphyrin(IX), gradually followed by spontaneous formation of a covalent bond between the polypeptide and porphyrin ring in the reconstituted apo-C14A. Further analysis suggested that the residual Cys11 and vinyl group of the porphyrin ring linked through the thiol-ene reaction promoted by light under ambient conditions. In this study, we describe the kinetic improvement of the covalent bond formation in accordance with the mechanism of the photoinduced thiol-ene reaction, which involves a thiyl radical as a reaction intermediate. Adding a radical generator to the reconstituted C14A mutant with either heme-*b* or zinc(II) porphyrin accelerated the bond-forming reaction, which supported the involvement of a radical species in the reaction. Partial observation of the reconstituted C14A in a dimer form and detection of sulfuryl radical by EPR spectroscopy indicated a thiyl radical on Cys11, a unique cysteinyl residue in *rC*_552_ C14A. The covalent bond forming mediated by the radical generator was also adaptable to the reconstituted apo-C14A with manganese(II)-protoporphyrin(IX), which also exhibits light-mediated covalent linkage formation. Therefore, the radical generator extends the versatility of producing *c*-type-like cytochrome starting from a metallo-protoporphyrin(IX) and the apo-C14A instantaneously.

## 1. Introduction

Cytochromes are members of general membrane-bound hemoproteins that carry out electron transport in a biological system. The first report of cytochrome was described by MacMunn in 1884 as a respiratory pigment [1]. However, the term ‘cytochrome’ was introduced by Keilin in 1925 by defining a group of intracellular heme-containing proteins that undergo an oxidationreduction reaction and exhibit intense absorption bands between 510–615 nm in the reduced state [2]. Depending on a cofactor present in the cytochrome, it is classified into four major groups, cytochrome *a*, *b*, *c,* and *d*, of which types *b* and *c* mostly occur in nature [3,4]. Out of these, only heme *c* is characterized by the presence of at least one covalent thioether bond between a cysteine of the protein and a vinyl group of the heme. All the other types, e.g., *a*, *b,* and *d* hemes, are not covalently bound to the protein, although they differ in the peripheral substitution of the heme [4]. In most cases, the heme attachment site of Cyt *c* consists of a Cys-X-X-Cys-His pentapeptide segment where the histidine residue serves as an axial ligand to the hexacoordinated iron, and the cysteine residues form the thioether bonds to two vinyl groups of the porphyrin and X can be any amino acid residues except cysteine. The details of the formation of the covalent linkage in biosynthesis between cysteine residues and vinyl substitution of heme are poorly understood [5,6,7,8]. However, it has been long believed that the covalent attachment of the heme to the apo-protein could take place only in the in vivo process through enzymatic assistance [9].

To investigate more on the covalent attachment, an *in vivo* conversion of cytochrome *b* into cytochrome *c* was achieved by Barker et al. [10,11]. They introduced two cysteine residues into the amino acid sequence of cytochrome *b*_562_ in positions homologous to those found in many cytochrome *c*. Overexpression of the cysteine mutant in *E. coli* yielded cytochrome *c,* where the cysteine residues were involved in thioether bonds with the heme. Similarly, Lin et al. reported the conversion of cytochrome *b*_5_ into a cytochrome *c*-like protein by introducing a cysteine residue close to the vinyl groups of the heme *b* [12]. They postulated that the cysteine-heme covalent linkage could be in the forms of either an ordinary thioether bond or [heme-CO-CH_2_-S-CH_2_-C_α_] depending on a distance between the cysteinyl thiol and a vinyl group. However, a mechanism of the unusual covalent linkage formation was not discussed in the report since the authors could not reproduce the same linkage *in vitro*.

The first *in vitro* formation of a covalent bond between a cysteine residue and heme was reported by Ferguson and his coworkers [13]. They demonstrated that apo-Cyt *c*_552_ from *Hydrogenobacter thermophilus* prepared by using a method of Fischer et al. [9] was able to incorporate the ferrous heme *b* and spontaneously form thioether bonds between the vinyl groups of the heme and cysteine residues [14,15,16]. However, the chemistry behind the thioether bond formation was not mentioned.

Previously, we reported a cytochrome *c* mutant (*rC*_552_ C14A) where the recombinant cytochrome *c*_552_ from *Thermus thermophilus* HB8 was engineered to be expressed and accumulated in the cytoplasm of *E. coli* host cell in addition to the replacement of Cys14 to Ala. The original heme species in *rC*_552_ C14A was removable by facile chemical treatments. The resultant polypeptide in the heme-free apo-form (apo-C14A) retained reactive Cys11 and compact conformation amenable to quantitative reconstitution with either native heme *b* or Zn-protoporphyrin(IX) (Zn-PPIX) [17]. Unusual high thermal stability and structural rigidity of Apo-C14A prompted us to do reconstitution with heme *b* and Zn-Protoporphyrin(IX), which resulted in the instant incorporation of artificial heme species. The reconstitution was followed by a similar spontaneous covalent linkage formation between Cys11 and a vinyl group of the incorporated heme species. Therefore, the formation of an artificial *c*-type cytochrome was achieved.

However, the conversion process was rather slow and took 24 h to finish the covalent linkage formation, making the creation of artificial *c*-type cytochrome a time-consuming process. In addition, in terms of the mechanistic approach, no descriptions were made. In the present study, we focused our attention on investigating in more detail the mechanistic aspect of the linkage formation and accelerating the process. It was found that the in-vitro conversion of type *b* heme into type *c* heme inside the C14A scaffold was achieved only in the presence of light. However, linkage formation is prohibited in the absence of light. Based on these findings, we postulated that the covalent linkage is essentially taking place through some click reaction between the thiol group of Cys11 and the vinyl group of the incorporated protoporphyrin species, which involved some thiol radical [18]. To establish the fact, a water-soluble radical generator is introduced with the expectation that the radical could enhance the formation rate of covalent linkage through the quick formation of the cysteine radical in the medium. Consistent with the assumption, the radical generator accelerated the reaction between the vinyl group of incorporated heme *b* species and Cysteine11 residue of the protein, and instant conversion of heme *b* into heme *c* was achieved within 2 min. The involvement of the Cysteine11 radical mediated through the radical generator was established by the isolation of the Cys11 radical with an EPR spectrum with an isotropic signal at *g* = 2.004. This supports the mechanistic pathway of the covalent linkage formation between heme *b* and Cysteine for converting heme *b* into heme *c*. The covalent linkage formation in the presence of a radical generator with a series of different metallo-protoporphyrins ends up with similar results. Therefore, the present study describes the first radical mediated instant *in vitro* formation of Cytochrome *c*-type protein starting from heme *b* with mechanistic details.

## 2. Results

### 2.1. Formation of a Covalent Linkage in Ferric or Ferrous Heme Reconstituted Cytochrome c552 (C14A) in the Presence of Light

**Figure 1:**
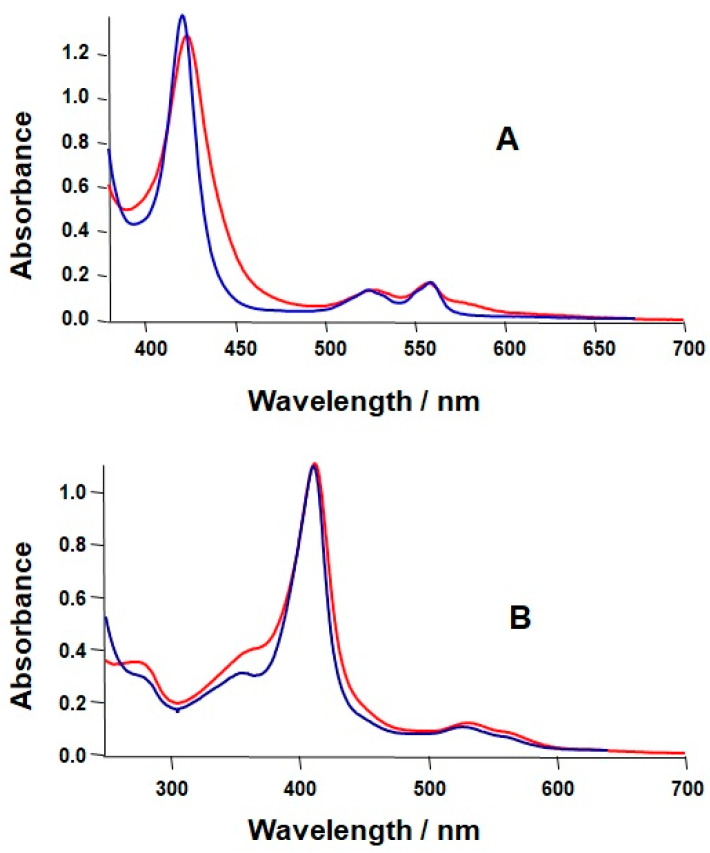
The UV-Vis spectra of the C14A mutant reconstituted with (**A**) ferrous and (**B**) ferric heme *b*. The spectra immediately after the reconstitution (red lines) show progressive blue shifts (blue lines) under ambient conditions.

**Figure 2:**
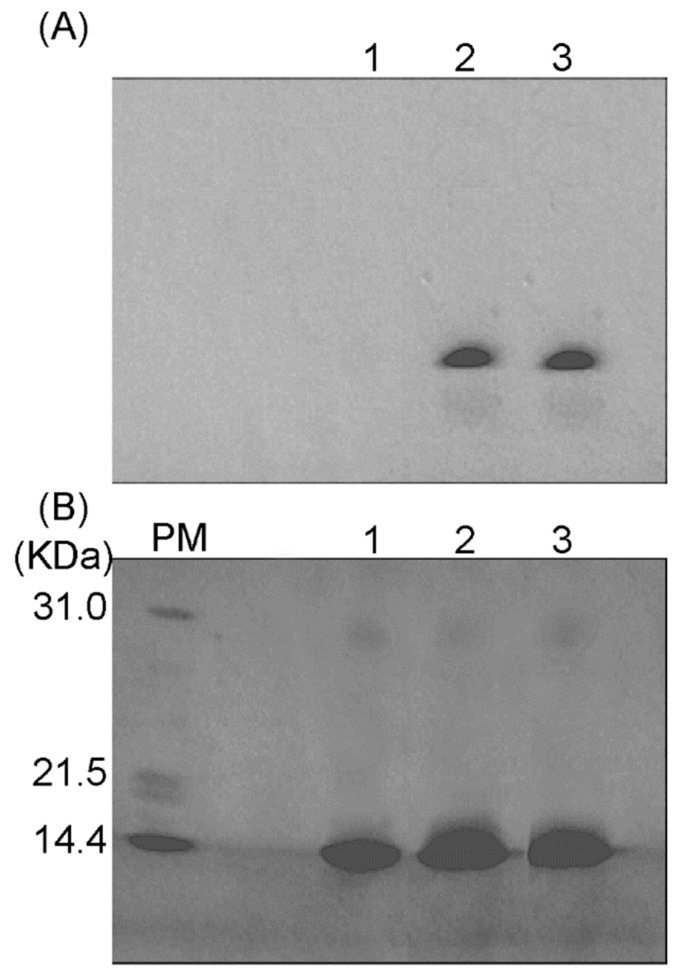
SDS-PAGE (15%) of apo-C14A (1) immediately after reconstitution with ferric heme, and reconstituted with (2) ferric and (3) ferrous heme *b* after showing the blue shifts in the UV-Vis spectra. (**A**) heme staining followed by (**B**) coomassie blue staining. PM: Protein marker.

After the reconstitution of apo-C14A with heme *b* species, ferrous cytochrome *b* showed a progressive blue shift in its UV–vis spectrum (Figure 1 and Table 1) for two days in the presence of light. A similar observation was reported when a thioether bond was formed between the heme and polypeptide in apo-*Ht* Cyt *c*_552_ reconstituted with the ferrous heme *b* [19]. In accordance with the report, the heme staining on SDS-PAGE gels for the resultant protein revealed a heme covalently bound to the polypeptide (Figure 2). The pyridine hemochrome spectrum showed the α-band at 553.0 nm, characteristic of a heme *b* derivative where only one cysteine is covalently connected with the heme through a single thioether bond resulting in the saturation of one of the two vinyl groups. [13] A thiol group in the resultant protein was not detected using an Ellman’s reagent. These results indicate that ferrous cytochrome *b* can form a covalent linkage between one of the two vinyl groups of the heme and thiol group of Cysteine11 (Cys11). Ferric cytochrome *b* also showed a spontaneous blue shift in the UV–vis spectrum (Figure 1B and Table 1). The heme staining (Figure 2), pyridine hemochrome spectrum (α-band at 553.0 nm), and Ellman’s reagent test supported the formation of the same covalent linkage in ferric cytochrome *b* as that observed in ferrous cytochrome *b*. This result is distinctive of the ferric cytochrome *b* obtained from apo-C14A as reported earlier [13,14,20].

### 2.2. Formation of a Covalent Linkage in Zn-PPIX Reconstituted C14A in the Presence of Light

**Figure 3:**
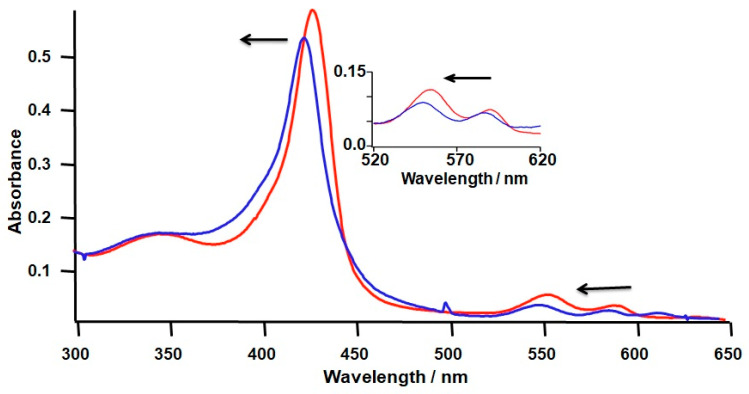
The spectral change of C14A reconstituted with Zn-PPIX. The UV-Vis spectrum immediately after the reconstitution (red line) shows spontaneous blue shift (blue line).

To establish the versatility of the covalent linkage formation in reconstituted C14A proteins for the formation of *c*-type cytochrome proteins, Zn-PPIX is incorporated in the apo-C14A scaffold. Following the incorporation of Zn-PPIX into apo-C14A (Zn-C14A), a progressive blue shift in the UV-Vis spectrum (from 430, 555, and 592 nm to 424, 551, and 586 nm, respectively) was observed (Figure 3) in the presence of light. A similar observation was described in a previous study on the reconstitution of *Ht* apo-Cyt *c*_552_ with Zn-PPIX. This was used as an index of the thioether bond formation between the peripheral vinyl groups and cysteinyl thiols in the polypeptide [21]. Under the present reaction conditions, it took 48 h to complete the reaction. However, in the absence of light, the conversion did not occur, even two weeks later.

### 2.3. Introduction of Radical Initiators

**Scheme 1:**
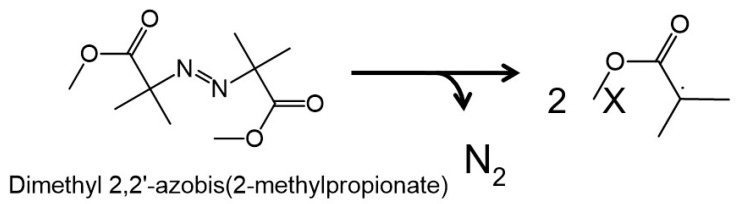
Dimethyl 2,2′-azobis(2-methylpropionate) used as a radical generator.

A detailed inspection of the *in vitro* covalent linkage process between the vinyl group of heme and Cys11 revealed that light is essential to promote the reaction for all heme species employed in this study. This finding implied that a photochemically generated cysteine radical initiates the reaction by attacking a vinyl group of the heme species. Analogous reactions were reported for photoinduced thiol-ene click chemistry [22,23]. To inspect the possibility of a radical-initiated reaction, we attempted a reaction of Zn-PPIX reconstituted C14A with an external radical generator. An initial choice was dimethyl 2,2′-azobis(2-methylpropionate) which generates a radical in solution by the liberation of nitrogen under weak UV light (365 nm) (Scheme 1) [24].

The radical generator promoted the covalent linkage formation between Zn-PPIX and Cys11 that finished within 1 h under the present reaction conditions. The UV-Vis spectrum and pyridine metallochrome spectrum suggested a saturation of one of the vinyl groups of Zn-PPIX, which was identical to the results obtained only in the presence of light. A product of the radical reaction showed a homogeneous band corresponding to Cyt *c*_552_ on the SDS-PAGE gel. Similar results were obtained for the reconstituted C14As with the ferrous and ferric heme *b*.

**Figure 4:**
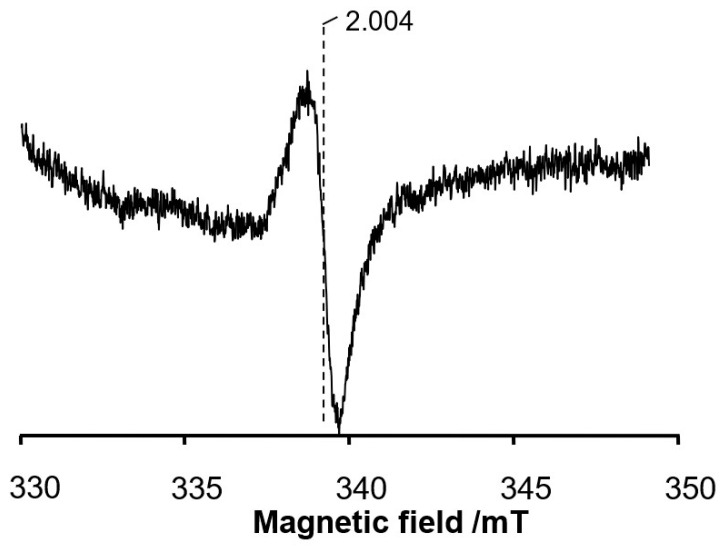
EPR spectrum of Zn-C14A mixed after the radical generator. The reaction solution was frozen 30 min after the mixing. The spectrum was recorded at 10 K. Microwave power 0.5 mW; Field modulation 0.2 mT; modulation frequency 100 kHz.

An EPR spectrum of Zn-C14A (50 µM) 30 min after the reaction with the radical generator showed an isotropic signal at *g* = 2.004 with a signal width of 1 mT (Figure 4). The signal is attributable to a radical predominantly localized on the thiol group of Cys11, a unique cysteinyl residue in the C14A mutant, because of the higher *g*-value than those observed for radicals located on other residues in proteins. The radicals on Trp, Tyr, His, and Phe residues showed signals in the range between *g* = 2.001 and 2.002 [25,26,27]. A control experiment was carried out with the Wild Type Cytochrome c_552_, where both cysteine residues already covalently connected with the natural heme, in the presence of the same radical initiator does not afford any relevant signal in the EPR spectrum under similar circumstances. The finding also indicates that the isotropic signal that appeared at *g* = 2.004 in the EPR spectrum originated from a non-other amino acid sequence than free cysteine moiety present in the C14A mutant.

**Figure 5:**
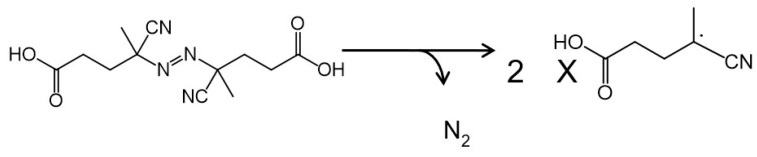
4,4′-Azobis(4-cyanovaleric acid) used as a water-soluble radical generator.

The acceleration of the covalent linkage formation in the presence of dimethyl 2,2′-azobis(2-methylpropionate) suggests that the formation of the cysteine radical is involved in the covalent linkage formation. The EPR spectrum of the reaction mixture supports this suggestion. A relatively slow rate to finish the linkage formation (1 h under present reaction condition) could be associated with sparing solubility of the employed dimethyl 2,2′-azobis(2-methylpropionate) to an aqueous solution. Consistent with the assumption, the use of a fully water soluble radical generator, 4,4′-Azobis(4-cyanovaleric acid) (Figure 5), in place of dimethyl 2,2′-azobis(2-methylpropionate) dramatically accelerated the linkage formation. When the newly employed radical generator (1 mM) was mixed with Zn-C14A (10 µM), a blue shift in the UV-Vis spectra was achieved in two minutes, indicating the instant conversion of Zn-C14A into *c*-type cytochrome through covalent linkage formation between Zn-PPIX and Cys11 in the same fashion as observed earlier in the presence of light. Again, no unfavorable effects of the radical generator were observed on the polypeptide of C14A. The resultant covalently linked engineered C14A mutants were subjected to CD spectrum, which followed a similar trend of gaining stability after the covalent linkage formation, as observed earlier. A significant improvement is observed in the thermal stability of the Zn-C14A upon covalent linkage formation, where the *T*_m_ is increased from 58.5 °C to 63.5 °C in the temperature-dependent CD spectra in 2 M guanidine.HCl studies, whereas only slight improvement was observed in the case of heme *b*-C14A (from 57.6 to 57.9 °C). (Supplementary Material, Figure S1).

### 2.4. Covalent Linkage Formation in Other Reconstituted Proteins

**Figure 6:**
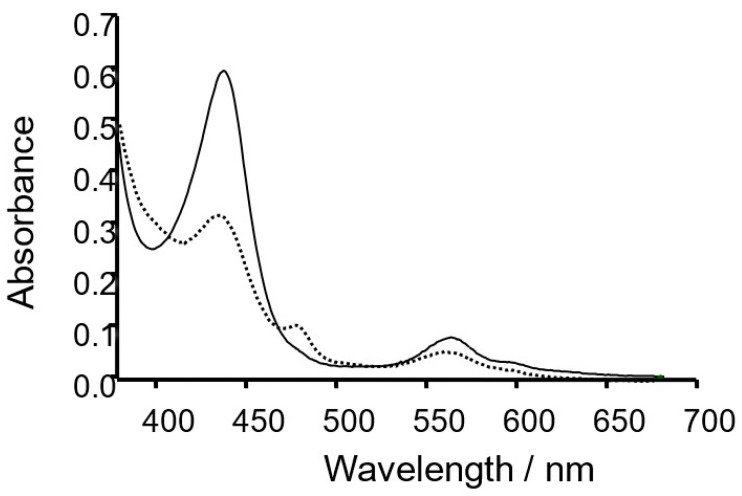
The UV-Vis spectral change of C14A reconstituted with Mn-PPIX (Mn-*rC*_552_) in reduced form. The UV-Vis spectrum immediately after the reconstitution (solid line) shows a spontaneous blue shift (dotted line) in the presence of light. An identical shift was observed after the reaction with an organic radical generator.

A successful reconstitution of Apo-C14A with Mn(III)-PPIX is achieved (Figure S2). Although the ferric and ferrous hemes incorporated in C14A similarly form covalent linkage, different oxidation states led to different results when Mn-PPIX was a prosthetic group of the reconstituted C14A. UV-Vis bands of Mn(II)-C14A showed a progressive blue shift (Figure 6 and Table 2) in the presence of light. Similar to Zn-C14A, the blue shift in the spectrum corresponded to the covalent linkage formation between Mn(II)-PPIX and Cys11, which was confirmed by acid-butanone extraction and the Ellman’s test. By contrast, in the case of Mn(III)-C14A, no covalent linkage formation between Mn(III)-PPIX and Cys11 took place despite extensive manipulation of reaction conditions. A similar covalent linkage was also observed in the absence of light when an organic radical generator was used, although the rate of the linkage formation in Mn(II)-C14A was significantly improved. However, in the case of Mn(III)-C14A, no covalent linkage was achieved ever under radical generator. Therefore, only Mn(II)-C14A can form the covalent linkage with Cys11 in the reconstituted C14A, irrespective of the acceleration methods of the reaction.

**Figure 7:**
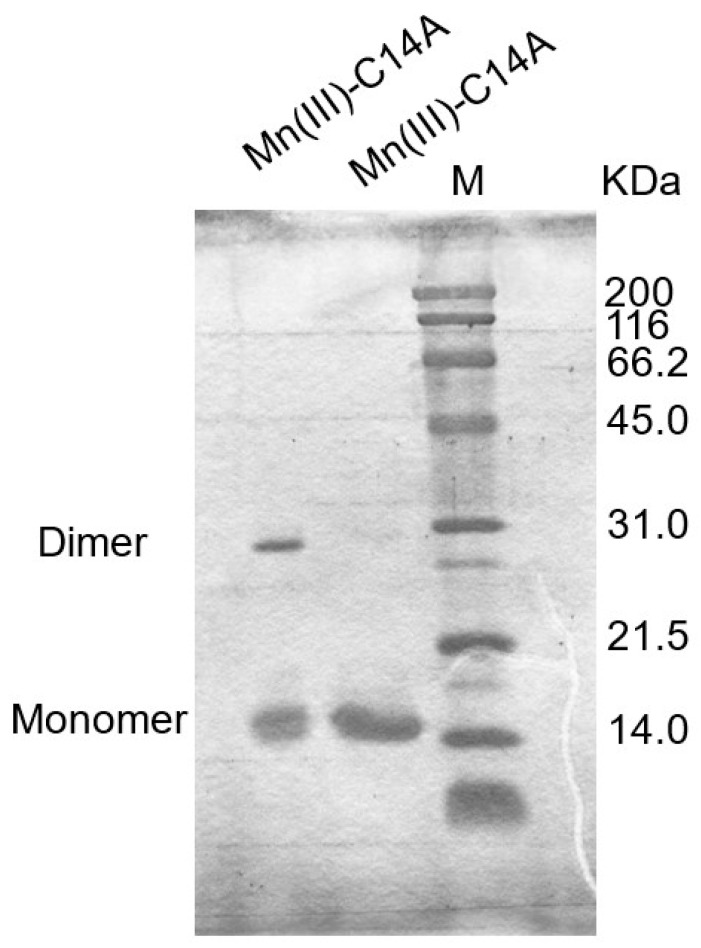
SDS-PAGE (15%) of Mn-C14A after the radical treatment. The Mn(II)-C14A shows a homogeneous band corresponding to the C14A monomer, whereas Mn(III)-C14A shows partial dimer formation. Samples were treated with SDS solution without containing a reducing agent, mercapto-ethanol. M: Protein Marker.

After reacting with the radical generator, Mn(III)-C14A showed dimer formation on the SDS-PAGE gel (without a reducing agent, mercapto-ethanol), which was not observed for Mn(II)-C14A (Figure 7). This is rationally described by intermolecular disulfide bond formation between sulfhydryl radicals on Cys11s, indicating that for a Cys11 radical, a neighboring vinyl group of Mn(III)-PPIX is less reactive than a sulfhydryl radical on the other Mn(III)-C14A. Interestingly, a redox reaction between Mn(II)-C14A and Mn(III)-C14A after the covalent linkage formation was fully reversible. An apparent vestige of conformational distortion in the protein scaffold was not found for Mn(III)-C14A having the covalent linkage. This implies that Mn(III)-PPIX also occupies a cavity of apo-C14A at a suitable position to form the covalent linkage with Cys11. Based on these considerations, incompetence of the Mn(III)-C14A at the covalent linkage formation is not due to the inability to produce a reactive cysteinyl radical, nor an apparent difference in positions of Mn(III)-PPIX and Mn(II)-PPIX in the C14A cavity. A study to resolve this problem is in progress.

Table 3 summarizes results of the covalent linkage reaction of C14A with some metal-PPIXs.

## 3. Discussion

### 3.1. Light Promoted Covalent Linkage Formation

**Scheme 2:**
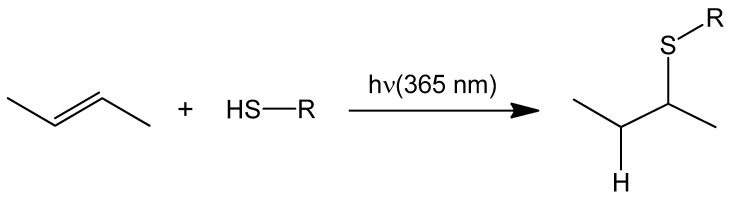
Thiol-ene click reaction.

As disused in our previous report [17], C14A immediately after the reconstitution contains intact Cys11 and a prosthetic group (ferric and ferrous hemes and Zn-PPIX) with the original vinyl groups without forming a covalent linkage. Therefore, the reconstituted C14As acted as simple cytochrome *b*-like proteins. However, prolonged observation showed that the reconstituted proteins underwent spontaneous covalent linkage formation between the prosthetic group and polypeptide. The acid-butanone treatment after the covalent linkage formation revealed the irremovable nature of the prosthetic group from the polypeptide. This established a transmutation of the prosthetic groups from the *b*-type to a *c*-type-like species. The disappearance of the cysteinyl thiol indicated that Cys11 is engaged in the covalent linkage formation with one of the vinyl groups in the prosthetic groups. Further investigations revealed that the covalent linkage formation required light, although the dependence of the reaction on a wavelength of light has not been determined at this moment. This finding reminded us of covalent linkage formation between a thiol and allene, namely a thiol-ene click reaction (Scheme 2), which was initially reported almost a hundred years ago [28]. Although a detailed mechanism of the reaction is still controversial, it has been proved that a thiol radical induced by UV light (365 nm) irradiation is essential to proceed with the reaction [22,23]. Many radical generators have been examined for this click reaction not only to improve the reaction rate but also to extend the adaptability of the reaction to various sets of thiols and enes [18,29,30]. Thus, an inspection of the effects of a radical generator on a possible thiol-ene reaction between the cysteinyl thiol and vinyl group was done.

### 3.2. Effects of a Radical Initiator on the Covalent Linkage Formation

In the present study, we selected 4,4′-Azobis(4-cyanovaleric acid) as a radical generator because of its high solubility in water and controlled release of radicals by ordinary fluorescent light irradiation, which may be harmless to the protein scaffold [24,31]. As expected, a significant acceleration was observed in the covalent linkage formation. In the case of C14A reconstituted with a ferrous heme, the reaction finished within 2 min. by using the radical generator, while the same reaction required 24 h to be completed when only light was used as a reaction promoter. Studies were carried out with all the reconstituted C14A prepared in the present study. As summarized in Table 3, the promotion of the linkage formation in the presence of light and the radical generator has the same restriction, suggesting that they proceed reactions through a common pathway that is presumably initiated at the radical thiol formation on Cys11. The hypothesis is supported by detecting a radical dominantly localized on a sulfur atom during the reaction with the radical generator.

The SDS-PAGE gel of Mn(II)-C14A and Mn(III)-C14A after the radical treatment focused more light on the proposed radical pathway (Figure 7). A fully covalently linked Mn(II)-C14A was obtained after the radical reaction. However, Mn(III)-C14A did not undergo the covalent linkage formation except for some dimer formation. A plausible interpretation of this observation is that the cysteinyl thiol radical intramolecularly reacts with the vinyl group of the Mn(II)-PPIX, giving no chance of any inter-protein reaction, while an inter-protein disulfide bridge is formed between two cysteinyl radicals to produce an Mn(III)-C14A dimer, due to inability of the cysteinyl radical to immediately attack a vinyl group of Mn(III)-PPIX.

### 3.3. Proposed Mechanism for the Covalent Linkage Reaction

**Scheme 3:**
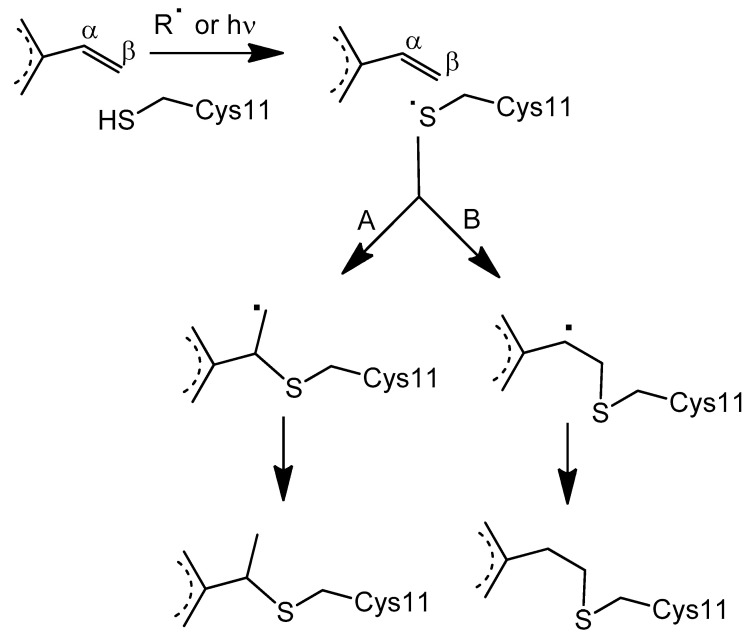
A plausible mechanism for the covalent linkage formation in reconstituted proteins in the presence of a radical initiator.

Based on the findings regarding the covalent linkage formation under different reaction conditions, a plausible mechanism using a radical generator is suggested, as shown in Scheme 3. The formation of the cysteinyl radical is promoted by a radical initiator (a radical generator or light). This cysteinyl radical attacks a vinyl group on the porphyrin ring to form a covalent linkage. Depending on the position of the radical attack (α or β carbon atom of the vinyl group), two different covalent linkages are possible (pathway A or B). However, pathway B would be more favorable as it passes through a much more stable secondary radical intermediate adjacent to the porphyrin ring. This is contrary to the in vivo formation of Cyt *c*, which has a thioether bond produced through the thermodynamically unfavorable pathway A in the current reaction scheme [4,5,32,33,34]. This highlights the unusual selectivity of the enzymatic thioether formation in Cyt *c*, which is believed to require 10 accessory proteins (namely Cyt *c* maturation proteins) [4,5,34].

### 3.4. EPR Signal Indicates the Involvement of Cysteine 11 Residue in Covalent Linkage Formation

An isotropic EPR signal at *g* = 2.004 appeared when reconstituted Zn-C14A reacted with the radical generator, whereas such a signal could not be observed when the same radical generator was added with a WT variant under the same experimental conditions. This observation is expected because, in the case of WT, both the cysteine residues in 11 and 14 positions engaged in the thioether bond with the heme moiety and therefore are not available to react with the radical generator to yield a relatively stable thiyl radical. These findings indicate the involvement of the free Cysteine 11 residue in the covalent linkage formation. However, the isotropic EPR signal that appeared in *g* = 2.004 is apparently contradictory to those reports mentioning a thiyl radical where the axially symmetric signals appeared at *g* = 2.008 [35,36]. Yet, recent interesting research reports an EPR signal that appeared at *g* = 2.004 by a tyrosine radical which involves a coupling with a cysteine residue as the signal could not be observed after the unavailability of cysteine residue, which indicated a formal transfer of a radical from the cysteine to tyrosine [37]. This is analogous to other previous reports where it was mentioned that the tyrosyl radical appeared at *g* = 2.004 [25,26]. Therefore, these results highly suggest possible involvements of other residues such as tyrosine through a coupling or radical exchange with the cysteine and/or an association of oxygen present in the reaction mixture resulting in a sulfuryl-kind of radical, which is well-reported in literature [38,39,40]. Nevertheless, there might be a possibility for cysteine, in the presence of an amino-acid environment and oxygen, to yield a secondary radical that is perhaps isolated in the EPR spectra. A detailed mechanistic and kinetic approach is underway and will hopefully be the subject of future work.

### 3.5. Oxidation State in a Metal Ion Which Affects the Linkage Formation

The covalent linkage formation could be achieved for Mn(II)-C14A, while in the case of Mn(III)-C14A, the covalent linkage formation was fully restricted. This phenomenon raised the idea that promoting the covalent linkage, orientation, access, and proximity of Cys11 to the vinyl group is important. An increase in the oxidation state of the metal ions may perturb the position of the Mn-PPIX plane in the C14A scaffold, which causes a shift of the vinyl group so as not to react with Cys11.

## 4. Materials and Methods

### 4.1. Chemicals

All chemicals were purchased from Nakarai Tesque, Wako Co. (Tokyo, Japan) and Sigma-Aldrich (Tokyo, Japan) and used without further purification.

### 4.2. Expression and Purification of rC_552_ C14A Mutant in E. coli

Construction of an expression system for *rC*_552_ C14A, expression, and purification methods were described in our previous report.

### 4.3. Spectroscopy

UV–Vis spectra were recorded on a Shimadzu UV-2400 PC spectrometer or a MultiSpec-1500 spectrometer from Shimadzu Co. (Kyoto, Japan) equipped with a temperature controller. Circular dichroism spectra were recorded on a J-720WN CD spectrometer from JASCO Co. (Tokyo, Japan) with a temperature controller. A thin optical cell of 1 mm path length containing 10 μM protein solution in succinic buffer was used for all the measurements. To determine the melting point, the temperature was raised linearly from 20 to 95 °C at a rate of 1 °C min^−1^. All data were recorded at 222 nm, corresponding to an absorption maximum of a negative Cotton effect by α-helices.

### 4.4. Heme Staining

A heme staining assay was performed according to a technique of Francis et al. with some modification. [41] *O*-dianisidine (0.2 g) was dissolved in 20 mL of acetic acid, followed by the addition of 160 mL mili-Q. The SDS-PAGE gel was washed with a 12.5% trichloroacetic acid solution for 30 min, followed by washing with Mili-Q water for 30 min. A mixture of 20 mL of 0.5 M sodium citrate buffer (pH 4.4), 0.4 mL of 30% H_2_O_2,_ and 180 mL of the *o*-dianisidine solution was poured over SDS-PAGE gel. The whole system was kept in a rotary shaker until bands developed.

### 4.5. Reaction with Radical Generators

To promote the covalent linkage formation, radical generators dimethyl 2,2′-azobis(2-methylpropionate) and 4,4′-Azobis(4-cyanovaleric acid)) were dissolved in a minimum amount of ethanol which was then diluted by mili-Q water to prepare 10 mM stock solutions. The covalent linkage reactions were performed in a mixture of a 1mM radical generator and 10 µM reconstituted C14A under an Ar atmosphere. After the reaction, the radical generator was removed from the solution by overnight dialysis.

### 4.6. EPR Measurements

EPR spectra were recorded on an EMX-Plus X-band CW-EPR (Bruker Co., Billerica, MA, USA) equipped with a cryostat (ESR900, Oxford Co., Oxford, UK) for temperature control. A stock solution of dimethyl 2,2′-azobis(2-methylpropionate) was added to Zn-C14A (50 µM) to the final concentration of 1 mM. The resultant solution was transferred into an EPR tube 30 min after the mixing. The reaction mixture was subsequently frozen by immersing the sample tube in liquid nitrogen. A control sample was prepared without Zn-C14A and with the Wild Type Cytochrome c_552_ under the same procedure.

## 5. Conclusions

A method for effective, quick, and quantitative covalent linkage formation between an external prosthetic group and polypeptide was developed in the reconstituted proteins. Results obtained here lead to the facile creation of functional *c*-type-like proteins bearing artificial prosthetic groups, although there is a restriction in the oxidation states of a central metal ion currently. A settlement of this problem would extend the method’s versatility and enhance the possibilities of the C14A scaffold to use as a biocatalyst or biomaterial. Reproduction of the original thioether bond found in natural Cyt *c* is another big target. A further focus in this study should be on the reproduction of the natural thioether bond in the reconstituted C14A without using the enzymatic reaction, which will evaluate the biological significance of Cyt *c* maturation proteins. This would give rise to arguments about the roles of the accessory proteins in in vivo synthesis of Cyt *c*.

## Figures and Tables

**Table 1:** Absorption maxima of C14A reconstituted with ferric and ferrous heme *b* recorded immediately after the reconstitution and after the covalent linkage formation.

	λ_max_/nm (*e*/cm^−1^·mM^−1^)	
	Ferric Heme *b*	Ferrous Heme *b*
Immediate after the reconstitution	413 (99)	424 (128)
	532 (11), 564(8)	528 (13), 558 (17)
After forming the covalent linkage	411 (98)	421 (137)
	527 (9)	524 (13), 559 (17)

**Table 2:** Absorption maxima of Mn(II)-C14A recorded immediately after the reconstitution and after the covalent linkage formation.

	λ_max_/nm	
	Soret Band	Q-Band
Immediate after the reconstitution	438	564
After forming the covalent linkage	435	559

**Table 3:** Formation of the covalent linkage with different reconstituted proteins under the conditions employed in this study. (✓) The linkage formation is achieved; (🗶) the linkage formation could not be achieved.

Reconstituted Protein/Metal Ions	O_2_/Light	Radical
Ferric heme-C14A	✓	✓
Ferrous heme-C14A	✓	✓
Zn-C14A	✓	✓
Mn(II)-C14A	✓	✓
Mn(III)-C14A	🗶	🗶

## Data Availability

The data presented in this study are available on request from corresponding author.

## Supplementary Materials

The following supporting information can be downloaded at: https://www.mdpi.com/article/10.3390/biom12101329/s1, Figure S1: Comparison of thermo-stability of reconstituted C14A in monitored by the temperature dependence of the CD spectra at 222 nm in the presence of 2M Guanidine HCl.; Figure S2: The spectrophotometric titration of apo-C14A to Mn(III)-PPIX.

## References

[1] Mac Munn C.A. (1886). VI. Researches on myohamatin and the histohæmatins. Philos. Trans. R. Soc. London.

[2] Keilin D. (1926). A comparative study of turacin and haematin and its bearing on cytochrome. Proc. R. Soc. Lond..

[3] Bowman S.E.J., Bren K.L. (2008). The chemistry and biochemistry of heme c: Functional bases for covalent attachment. Nat. Prod. Rep..

[4] Kranz R.G., Richard-Fogal C., Taylor J.-S., Frawley E.R. (2009). Cytochrome c biogenesis: Mechanisms for covalent modifications and trafficking of heme and for heme-iron redox control. Microbiol. Mol. Biol. Rev..

[5] Barker P.D., Ferguson S.J. (1999). Still a puzzle: Why is haem covalently attached in c-type cytochromes?. Structure.

[6] Schulz H., Hennecke H., Thöny-Meyer L. (1998). Prototype of a heme chaperone essential for cytochrome c maturation. Science.

[7] Kleingardner J.G., Bren K.L. (2015). Biological significance and applications of heme c proteins and peptides. Acc. Chem. Res..

[8] Pettigrew G.W., Moore G.R. (2012). Cytochromes C: Biological Aspects.

[9] Fisher W.R., Taniuchi H., Anfinsen C.B. (1973). On the role of heme in the formation of the structure of cytochrome c. J. Bio. Chem..

[10] Sawyer E.B., Stephens E., Ferguson S.J., Allen J.W., Barker P.D. (2010). Aberrant attachment of heme to cytochrome by the Ccm system results in a cysteine persulfide linkage. J. Am. Chem. Soc..

[11] Barker P.D., Nerou E.P., Freund S.M.V., Fearnley I.M. (1995). Conversion of cytochrome b562 to c-type cytochromes. Biochemistry.

[12] Lin Y.-W., Wang W.-H., Zhang Q., Lu H.-J., Yang P.-Y., Xie Y., Huang Z.-X., Wu H.-M. (2005). Converting Cytochrome b5 into Cytochrome c-Like Protein. ChemBioChem.

[13] Daltrop O., Allen J.W.A., Willis A.C., Ferguson S.J. (2002). In vitro formation of a c-type cytochrome. Proc. Natl. Acad. Sci. USA.

[14] Daltrop O., Ferguson S.J. (2003). The in Vitro Reactions Of Horse Heart Apocytochromec And Paracoccus Denitrificans Apocytochromec550 with Heme. J. Biol. Chem..

[15] Stevens J.M., Daltrop O., Allen J.W.A., Ferguson S.J. (2004). C-type cytochrome formation: Chemical and biological enigmas. Acc. Chem. Res..

[16] Metcafle C.L., Daltrop O., Ferguson S.J., Raven E.L. (2007). Tuning the formation of a covalent haem–protein link by selection of reductive or oxidative conditions as exemplified by ascorbate peroxidase. Biochem. J..

[17] Ibrahim S.M., Nakajima H., Ohta T., Ramanathan K., Takatani N., Naruta Y., Watanabe Y. (2011). Cytochrome c 552 from Thermus thermophilus engineered for facile substitution of prosthetic group. Biochemistry.

[18] Hoyle C.E., Bowman C.N. (2010). Thiol–ene click chemistry. Angew. Chem. Int. Ed..

[19] Daltrop O., Smith K.M., Ferguson S.J. (2003). Stereoselective in vitro formation of c-type cytochrome variants from Hydrogenobacter thermophilus containing only a single thioether bond. J. Biol. Chem..

[20] Barker P.D., Ferrer J.C., Mylrajan M., Loehr T.M., Feng R., Konishi Y., Funk W.D., Macgillivray R.T.A., Mauk A.G. (1993). Transmutation of a heme protein. Proc. Natl. Acad. Sci. USA.

[21] Daltrop O., Ferguson S.J. (2004). In vitro studies on thioether bond formation between Hydrogenobacter thermophilus apocytochrome c552 with metalloprotoporphyrin derivatives. J. Biol. Chem..

[22] Griesbaum K. (1970). Problems and possibilities of the free-radical addition of thiols to unsaturated compounds. Angew. Chem. Int. Ed..

[23] Zard S.Z. (2003). Radical Reactionsin Organic Synthesis.

[24] López-Alarcón C., Fuentes-Lemus E., Figueroa J.D., Dorta E., Schoeneich C., Davies M.J. (2020). Azocompounds as generators of defined radical species: Contributions and challenges for free radical research. Free Radic. Biol. Med..

[25] Miner K.D., Pfister T.D., Hosseinzadeh P., Karaduman N., Donald L.J., Loewen P.C., Lu Y., Ivancich A. (2014). Identifying the elusive sites of tyrosyl radicals in cytochrome c peroxidase: Implications for oxidation of substrates bound at a site remote from the heme. Biochemistry.

[26] Stubbe J., Van Der Donk W.A. (1998). Protein radicals in enzyme catalysis. Chem. Rev..

[27] Bolman P.S.H., Safarik I., Stiles D.A., Tyerman W.J.R., Strausz O.P. (1970). Electron paramagnetic resonance spectra of some sulfur-containing radicals. Can. J. Chem..

[28] Posner T. (1905). Beiträge zur Kenntniss der ungesättigten Verbindungen. II. Ueber die Addition von Mercaptanen an ungesättigte Kohlenwasserstoffe. Chem. Ber..

[29] Hoyle C.E., Lowe A.B., Bowman C. (2009). Thiol-click chemistry: A multifaceted toolbox for small molecule and polymer synthesis. Chem. Soc. Rev..

[30] Killops K.L., Campos L.M., Hawker C.J. (2008). Robust, efficient, and orthogonal synthesis of dendrimers via thiol-ene “click” chemistry. J. Am. Chem. Soc..

[31] Niki E. (2021). Commentary on “Azocompounds as generators of defined radical species: Contributions and challenges for free radical research” by López-Alarcón et al. Free Radic. Biol. Med..

[32] Allen J.W.A., Daltrop O., Stevens J.M., Ferguson S.J. (2002). C-type cytochromes: Diverse structures and biogenesis systems pose evolutionary problems. Philos. Trans. R. Soc. London B.

[33] Harvat E.M., Daltrop O., Sobott F., Moreau M., Barker P.D., Stevens J.M., Ferguson S.J. (2011). Metal and redox selectivity of protoporphyrin binding to the heme chaperone CcmE. Metallomics.

[34] Thöny-Meyer L. (2002). Cytochrome c maturation: A complex pathway for a simple task?. Biochem. Soc. Trans..

[35] Lassmann G., Kolberg M., Bleifuss G., Gräslund A., Sjöberg B.M., Lubitz W. (2003). Protein thiyl radicals in disordered systems: A comparative EPR study at low temperature. Phys. Chem. Chem. Phys..

[36] Kolberg M., Bleifuss G., Gräslund A., Sjöberg B.M., Lubitz W., Lendzian F., Lassmann G. (2002). Protein thiyl radicals directly observed by EPR spectroscopy. Arch. Biochem. Biophys..

[37] McCaslin T.G., Pagba C.V., Hwang H., Gumbart J.C., Chi S.H., Perry J.W., Barry B.A. (2019). Tyrosine, cysteine, and proton coupled electron transfer in a ribonucleotide reductase-inspired beta hairpin maquette. Chem. Commun..

[38] Sevilla M.D., Yan M.Y., Becker D. (1988). Thiol peroxyl radical formation from the reaction of cysteine thiyl radical with molecular oxygen: An ESR investigation. Biochem. Biophys. Res. Commun..

[39] Sevilla M.D., Becker D., Swarts S., Herrington J. (1987). Sulfinyl radical formation from the reaction of cysteine and glutathione thiyl radicals with molecular oxygen. Biochem. Biophys. Res. Commun..

[40] Nauser T., Koppenol W.H., Schöneich C. (2015). Protein thiyl radical reactions and product formation: A kinetic simulation. Free Radic. Biol. Med..

[41] Francis R.T., Becker R.R. (1984). Specific indication of hemoproteins in polyacrylamide gels using a double-staining process. Anal. Biochem..

