# Supplementary material for: Radical Mediated Rapid In Vitro Formation of c-Type Cytochrome
Source: Biomolecules. 2022 Sep 20;12(10):1329. doi: 10.3390/biom12101329 (PMC9599503; doi:10.3390/biom12101329)
Supplement: Supplementary file 1 [file biomolecules-12-01329-s001.zip › biomolecules-1897278-supplementary.pdf]

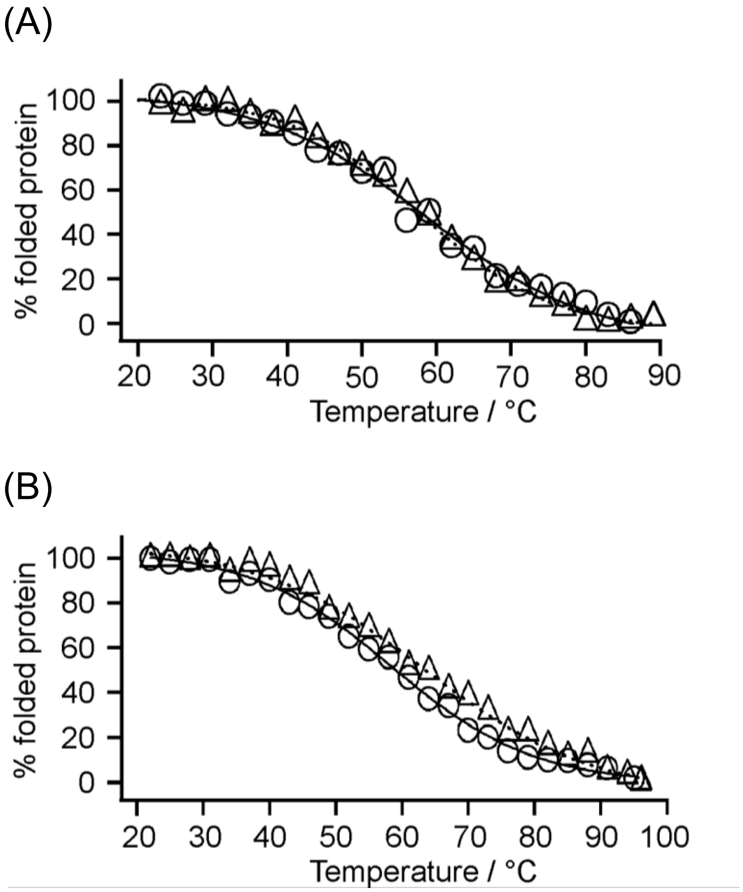

Figure S1. Comparison of thermo-stability of reconstituted C14A in monitored by the temperature dependence of the CD spectra at 222 nm in the presence of 2M Guanidine•HCl. (A) Reconstituted C14A with ferric heme *b* before (○) and after (Δ) the covalent linkage formation, (B) reconstituted C14A with Zn-PPIX before (○) and after (Δ) the covalent linkage formation

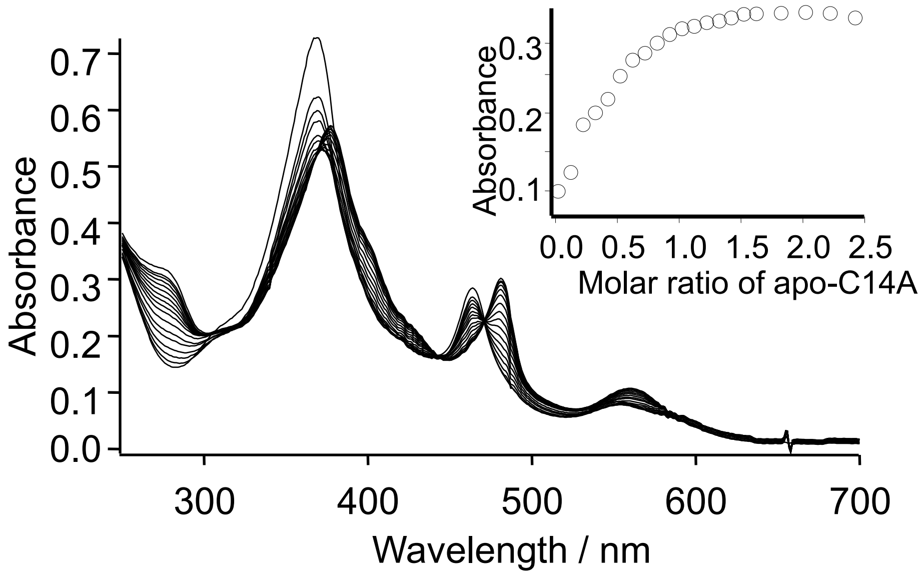

Figure S2. The spectrophotometric titration of apo-C14A to Mn(III)-PPIX. The apo-protein is reconstituted with Mn(III)-PPIX to yield a corresponding Mn-substituted cytochrome. The inset displays a profile of the titration monitored at the emerging soret band (480 nm) of the cytochrome.
